# Bruton’s tyrosine kinase inhibition for the treatment of allergic disorders

**DOI:** 10.1016/j.anai.2024.03.002

**Published:** 2024-03-14

**Authors:** Erica V. Lin, Ragha V. Suresh, Melanie C. Dispenza

**Affiliations:** 1 Johns Hopkins University School of Medicine, Department of Medicine, Division of Allergy and Clinical Immunology, Baltimore, MD, USA; 2 Center for Drug Evaluation and Research, United States Food and Drug Administration, Silver Spring, MD, USA

**Keywords:** Allergy, anaphylaxis, Bruton’s tyrosine kinase, chronic urticaria, IgE

## Abstract

IgE signaling through its high-affinity receptor FcεRI is central to the pathogenesis of numerous allergic disorders. Oral inhibitors of Bruton’s tyrosine kinase (BTKis), which are currently FDA-approved for the treatment of B cell malignancies, broadly inhibit the FcεRI pathway in human mast cells and basophils and therefore may be effective allergen-independent therapies for a variety of allergic diseases. The application of these drugs to the allergy space was previously limited by the low kinase selectivity and subsequent toxicities of early-generation compounds. Fortunately, next-generation, highly-selective BTKis in clinical development appear to have more favorable risk-benefit profiles, and their likelihood of being FDA-approved for an allergy indication is increasing. Recent clinical trials have demonstrated the remarkable and rapid efficacy of the second-generation BTKi acalabrutinib in preventing clinical reactivity to peanut ingestion in peanut-allergic adults. Additionally, next-generation BTKis including remibrutinib effectively reduce disease activity in patients with antihistamine-refractory chronic spontaneous urticaria. Finally, several BTKis are currently under investigation in early clinical trials for atopic dermatitis and asthma. In this review, we summarize recent data supporting the use of these drugs as novel therapies in food allergy, anaphylaxis, urticaria, and other allergic disorders. We also discuss safety data derived from trials utilizing both short-term and chronic dosing of BTKis.

## INTRODUCTION

IgE and its signaling pathway play key roles in the pathogenesis of numerous allergic disorders. Binding of specific IgE to its allergen cross-links the high-affinity receptor FcεRI on the surface of mast cells and basophils, initiating a signaling cascade which culminates in cellular degranulation (releasing histamine and other mediators), leukotriene and prostaglandin production, and cytokine synthesis. These released mediators cause the signs and symptoms of allergic disorders and are among the primary targets of current allergy pharmacotherapy, especially for treating acute allergic responses. Unfortunately, there are no therapies that reliably prevent IgE-mediated reactions, including corticosteroids. IgE-targeting biologics including omalizumab can improve symptoms of chronic urticaria and asthma through reducing availability of free IgE in circulation, but cannot completely suppress IgE-mediated activation of human mast cells and basophils. Therefore, there is an unmet need for novel therapies capable of preventing IgE-mediated allergic reactions.

**Figure 1:**
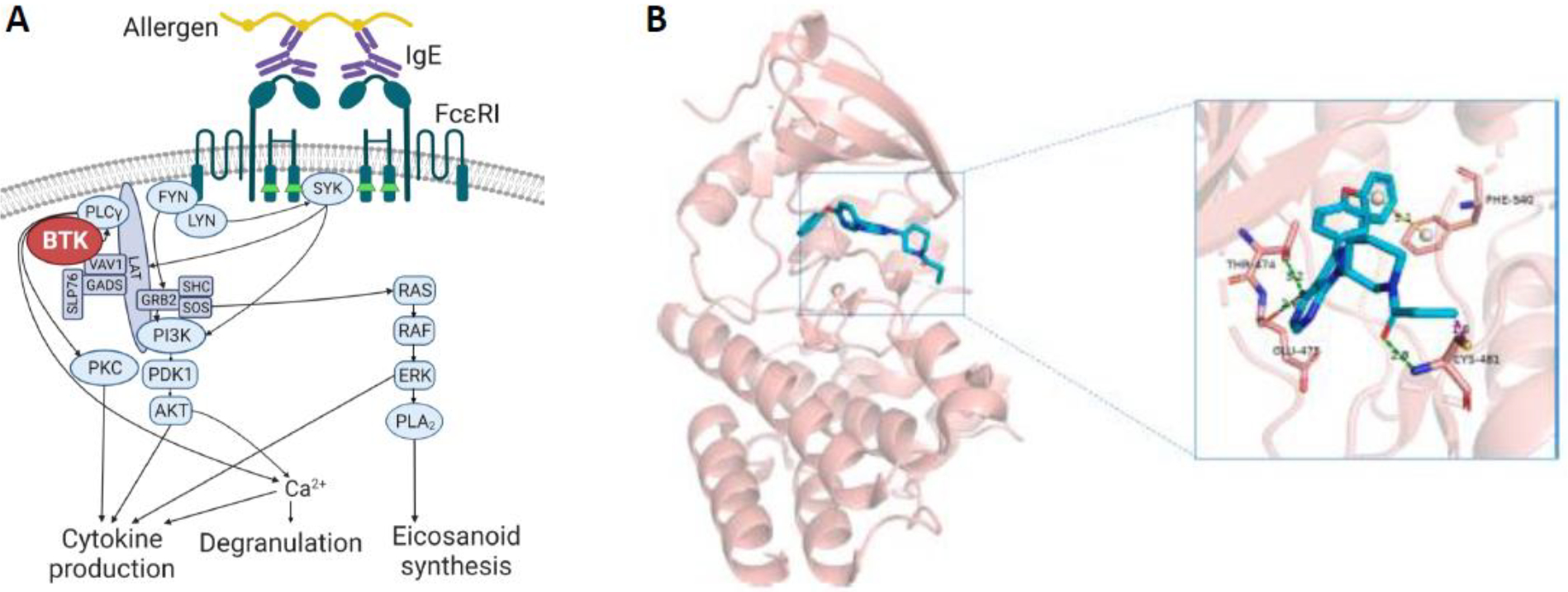
BTK structure and function. (A) BTK is a cytoplasmic non-receptor tyrosine kinase expressed primarily in leukocytes. It is an essential enzyme in the IgE-mediated activation of human mast cells and basophils through the high-affinity receptor FcεRI, resulting in cellular activation, degranulation, and production of prostaglandins, leukotrienes, and inflammatory cytokines. Figure created with BioRender. (B) The currently approved BTKis target residue C-481 in the kinase domain (box call-out) to inhibit enzymatic activity. Figure reproduced with permission from Wang et al^85^.

Bruton’s tyrosine kinase (BTK) is an essential enzyme for signaling through the FcεRI pathway in human mast cells and basophils (Figure 1A). Therefore, BTK is a major potential target for preventing IgE activation of these cells by any allergen, whether it be food, drug, venom, or aeroallergen. Though they are FDA-approved for B cell malignancies, unacceptable toxicities of the first- and second-generation BTK inhibitors (BTKis) have limited their application to non-cancer indications. Fortunately, the next-generation BTKis in development demonstrate a favorable side effect profile even with chronic use^1^, opening numerous possibilities for the use of these drugs to treat various allergic disorders. This review discusses recent clinical trials utilizing BTKis for the prevention of acute food-induced anaphylaxis, chronic spontaneous urticaria (CSU), atopic dermatitis (AD), drug allergy, and asthma, as well as safety data for currently-approved BTKis and those in the pipeline.

## PHARMACOLOGY

BTK is a cytoplasmic non-receptor tyrosine kinase expressed primarily in leukocytes. In human mast cells and basophils, it is essential for signaling through FcεRI. A more detailed review of BTK signaling in allergic disorders has recently been published^2^. BTK is also important for other immunoglobulin receptor signaling pathways, including FcγR and the B cell receptor (BCR). As a result, pharmacologic BTK inhibitors were first developed for the treatment of B cell malignancies to suppress malignant B cell proliferation. Currently, there are four FDA-approved BTKis in the United States (Table 1). The first-in-class BTKi ibrutinib (manufacturers Janssen, Pharmacyclics, and AbbVie) is an oral, covalent, small molecule inhibitor that was first approved for mantle cell lymphoma in 2013 and is now approved for several other indications, including various lymphomas, leukemias, and chronic graft-versus-host-disease (cGVHD). Ibrutinib targets the cysteine-481 residue in the ATP-binding pocket of the BTK kinase domain, thereby preventing phosphorylation of downstream proteins (Figure 1B). Due to high conservation of the cysteine-481 binding site among multiple other cysteine-containing kinases, including the TEC family, early-generation BTKis including ibrutinib possess off-target activity of varying degrees. In particular, ibrutinib has activity on TEC kinase, EGFR, ITK, and several other kinases, which together likely account for its more severe side effect profile that includes bleeding, cardiac arrhythmia, hypertension, infection, and skin toxicities (see section on Safety Considerations below)^3^.

Acalabrutinib (Acerta and AstraZeneca) was the first of the second-generation compounds to be approved in 2017, followed by zanubrutinib (Beigene) in 2019. Both are covalent inhibitors with a similar binding site as ibrutinib, but with higher selectivity and fewer off-target effects^4,5^. Acalabrutinib has lower affinity for TEC kinase and does not have activity on EGFR, SRC-family kinases, and several others inhibited by ibrutinib. Conversely, pirtobrutinib (Eli Lilly) is a second-generation non-covalent inhibitor that was specifically designed to treat cancers that have become refractory to other BTKis^6^. Unlike its predecessors that only inhibit wildtype BTK, pirtobrutinib has additional activity on mutant BTK with cysteine-481 substitution mutations, which are the most common acquired mutations that confer cancer resistance to BTKis. All second-generation compounds appear to have more favorable toxicity profiles due to fewer off-target effects compared to ibrutinib^4,5,7–9^.

Next-generation BTKis in development for chronic urticaria and autoimmune diseases have further increased selectivity for BTK and possess improved safety profiles due to differential activity on BTK conformations^1^. Remibrutinib (LOU064, Novartis) is a covalent inhibitor with an alternative binding site on BTK, resulting in higher affinity for the inactive conformation. Because this binding site is unique to BTK (unlike the cysteine-481 site, which is common to numerous other kinases), remibrutinib achieves sustained BTK inhibition with lower off-target effects^10,11^. Additionally, rilzabrutinib (PRN1008, Principia Pharma/Sanofi) is a highly-selective covalent inhibitor with prolonged BTK occupancy due to a slower dissociation rate^12,13^. Overall, the newer BTKis in development have promising safety profiles with chronic use, expanding the possibilities for chronic use of BTKis for allergic disorders^1^.

Given the importance of BTK in the FcεRI pathway in human cells, it is not surprising that BTKis have proven to be effective inhibitors of IgE signaling, regardless of allergen or receptor cross-linking mechanism. Early studies showed that ibrutinib is remarkably effective in preventing IgE-mediated basophil activation and degranulation *in vitro* and *in vivo*^14^. *Ex vivo* basophil activation has even been used as a surrogate marker for *in vivo* drug occupancy of BTK in peripheral blood mononuclear cells, including in ibrutinib’s phase 1 clinical trial^15^. We and others have shown that brief exposures to BTKis *in vitro* prevent IgE-mediated activation of both human mast cells and basophils, thus preventing downstream release of histamine and β-hexosaminidase as well as production of prostaglandin D2 and inflammatory cytokines^14,16–18^. As demonstrated by studies in mice and humans, BTKis appear to have a rapid onset of action, within minutes *in vitro* and within hours to days *in vivo*^16,19^. Due to differences in mast cell and basophil lifespan, BTKis’ duration of efficacy for preventing systemic allergic reactions *in vivo* may potentially depend on the type of binding (covalent versus non-covalent), but studies suggest that their efficacy wanes within days regardless of inhibitor type. Basophils, which are continuously regenerated from bone marrow progenitors, may rapidly overcome the effects of a covalent inhibitor within days^15,20^. Although tissue-resident mast cells are thought to be long-lived *in vivo*^21^, covalent BTKis nonetheless lose inhibitory activity in human primary mast cells within one to two days after washout *in vitro*, suggesting that cultured mast cells produce new BTK protein within that timeframe^16^. Overall, these data demonstrate exciting possible applications of BTKis for allergic disorders, especially those that are IgE-mediated and/or require rapid therapeutic response or transient efficacy.

## ALLERGY INDICATIONS FOR BTK INHIBITORS

As proof of concept that BTKis can suppress mast cell and basophil activation in humans *in vivo*, we completed two pilot studies demonstrating that BTKis suppress skin prick test (SPT) responses to allergens as well as basophil activation. The first was an observational study in two patients who were prescribed ibrutinib as part of their treatment plan for their pre-existing malignancy^22^. At baseline, both patients had positive skin tests to aeroallergens (cat and ragweed). One week after starting ibrutinib, their aeroallergen SPTs had become negative, an effect sustained even one to two months later while still taking ibrutinib therapy. These results informed the design of a subsequent open-label trial, wherein we investigated the effects of ibrutinib on food SPT size and basophil activation testing in healthy adults with IgE-mediated peanut and tree nut allergies (NCT03149315)^20^. We enrolled six subjects with IgE-mediated allergy to peanuts and/or tree nuts (four of whom had histories of anaphylaxis after exposure), and administered a seven-day course of 420 mg oral ibrutinib once daily. All SPTs to foods (*n*=25 foods total) were suppressed within two days of starting ibrutinib therapy, with an average wheal area reduction of 77%. 44% of all SPTs became negative. Additional doses of ibrutinib up to seven days did not further suppress SPT size, suggesting that peak efficacy occurred within the two-day timepoint. As expected, *ex vivo* IgE-mediated basophil activation was wholly suppressed on ibrutinib therapy. No observable side effects or toxicities were observed with up to seven days of ibrutinib treatment. The results of these early studies have since informed subsequent trial design for the use of BTKis in allergic disorders.

### Anaphylaxis:

The most groundbreaking potential application of BTKis is for rapid-onset prevention of IgE-mediated anaphylaxis, which is a potentially life-threatening systemic allergic reaction to foods, drugs, stinging insect venoms, or other substances. There are no known therapies that can rapidly and reliably prevent anaphylaxis, and intramuscular epinephrine is the only FDA-approved therapy for its treatment, to be administered after the onset of reaction. Even with prompt and comprehensive medical treatment, anaphylaxis still causes hundreds of deaths per year in the United States^23^. Recent studies now suggest that BTKis could effectively prevent IgE-mediated anaphylaxis with rapid onset of action. While clinical trials have been only been conducted in food allergy, it is presumed that similar efficacy could be achieved in drug or venom allergy given the common mechanism of IgE-mediated systemic reactions.

**Figure 2:**
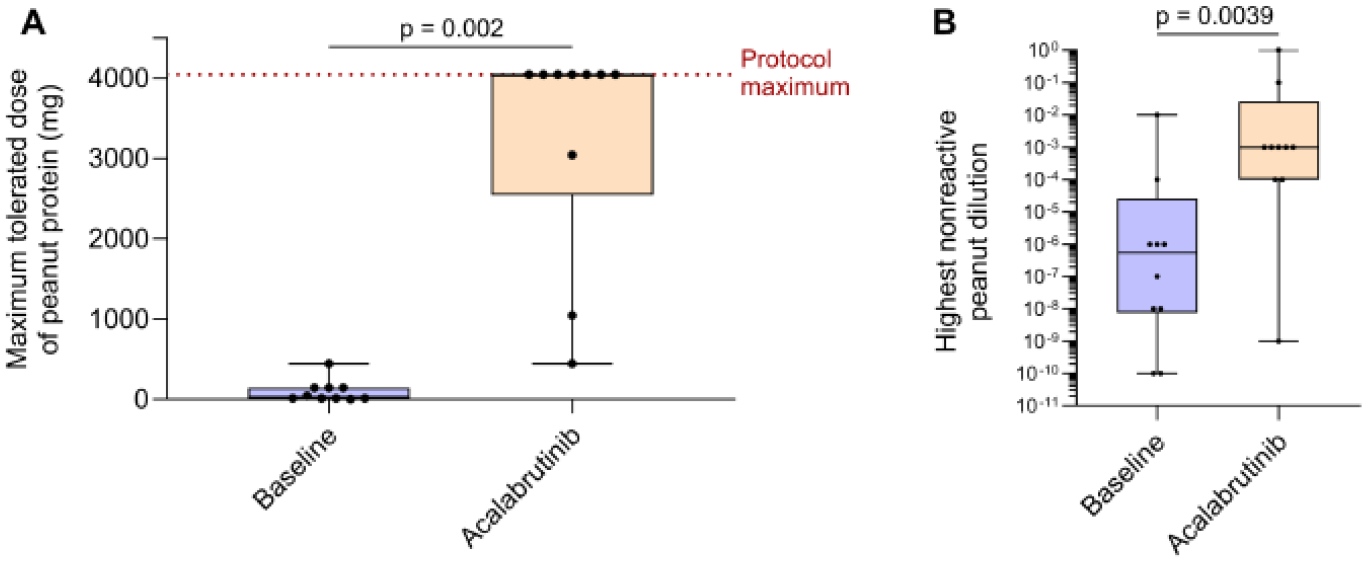
A two-day course of acalabrutinib prevents clinical reactivity to peanut ingestion during OFC in the majority of peanut-allergic adults. (A) After taking four 100 mg oral doses (a two-day course) of acalabrutinib, peanut-allergic adult patients experienced a significant increase in the amount of peanut that they could tolerate during oral food challenge from a median of 29 mg of peanut protein at baseline to 4,044 mg (the protocol maximum) during treatment. (B) The dilution of peanut extract that elicited a positive SPT response also significantly increased by several logs from baseline. Figures reproduced with permission from Suresh et al^19^.

We recently completed an open-label phase 2 clinical trial to test the efficacy of acalabrutinib in preventing clinical reactivity to peanut in peanut-allergic adults (NCT05038904)^19^. We enrolled 10 subjects with IgE-mediated allergy to peanut, confirmed by clinical history plus positive serum peanut-specific IgE and SPT to peanut extract. All subjects underwent a placebo-controlled graded oral challenge to peanut to determine their reactive dose. They were then allotted at least six weeks’ recovery time, after which they were given four standard doses of 100 mg acalabrutinib twice daily, returning on the morning of their fourth dose for repeat peanut challenge. At baseline, subjects tolerated a median of 29 mg (range 1 to 444 mg) of peanut protein without objective clinical reaction, as assessed using a modified PRACTALL scale^24^ (Figure 2A). During their subsequent oral challenge while taking acalabrutinib, seven of ten subjects tolerated 4,044 mg of peanut protein (the equivalent of about 16–20 peanuts), which was the maximum amount of peanut allowed in the study protocol, with no objective clinical reaction^19^. The remaining three subjects’ peanut tolerances increased from 14 mg at baseline to 444, 1,044, and 3,044 mg, respectively, while on acalabrutinib. In line with their increased tolerance, subjects demonstrated reduced skin mast cell sensitivity during end-point titration SPT to peanut extract, as shown by a significant increase in the highest non-reactive peanut dilution (Figure 2B). In terms of safety, four adverse events that could possibly or probably be related to treatment were observed: one grade 2 neutropenia (2.65 to 1.22 K/mm3), one grade 1 hemoglobin decrease (by 0.3 g/dL), and one grade 1 eosinophilia (absolute eosinophil count from 250/mm^3^ to 890/mm^3^), all of which were observed during acalabrutinib therapy and resolved by follow-up, as well as one grade 1 increase in liver function tests which occurred four weeks post-cessation of therapy. Side effects were largely transient, and none were associated with clinical events or symptoms. Total immunoglobulin levels and specific IgE levels remained stable during treatment. This study suggests that the current BTKis, while less selective than those in the pipeline, may still be used in short courses for allergy indications with minimal adverse effects.

These remarkable results suggest that BTKis can prevent clinical reactivity to foods in allergic patients. Given that all patients in the trial were able to consume over 300 mg of peanut protein without clinical reaction while taking acalabrutinib, it seems likely that standard doses of BTKis used for malignancies can confer protection against allergic reactions that result from small amounts of food allergens, as with consumption during an accidental food exposure. Furthermore, because most subjects in the trial consumed the maximum peanut amount without any objective reaction, it may be possible to utilize BTKis to reduce reactions after exposures to larger amounts of allergens, such as to facilitate drug desensitizations (discussed in the Drug Allergy section). Though not explicitly studied in humans, BTKis have also been shown to significantly protect against mortality during passive systemic anaphylaxis in humanized mice, which have mature human mast cells and basophils^16^. We demonstrated that as compared to vehicle, two oral human-equivalent doses administered 16 and 4 hours prior to bolus intravenous allergen challenge significantly reduced mortality in humanized mice (13% mortality in acalabrutinib group versus 39% in vehicle group)^16^.

Further studies are needed to determine factors that may predict the degree of BTKi efficacy, as the peanut allergy trial could not identify distinct patient characteristics that correlated with clinical response, including total or peanut-specific IgE levels, baseline basophil or skin test responses, body weight, or baseline tryptase level^19^. Interestingly, some insight may be gleaned from acalabrutinib’s incomplete inhibition of patients’ SPTs despite complete suppression of their basophils. This discrepancy suggests that the currently-approved acalabrutinib dose, which was optimized to target peripheral blood mononuclear cells including malignant B cells in circulation, is not sufficient for complete inhibition of tissue-resident mast cells. Pharmacodynamic assessments of acalabrutinib’s skin penetrance were not performed in early clinical trials, because they were not relevant for treating B cell malignancies. Another larger phase 2 dose-finding trial using remibrutinib in peanut-allergic adults is currently ongoing (NCT05432388) and will help determine the minimal BTKi doses needed to suppress SPTs, basophil activation, and overall clinical reactivity, and whether or not these doses differ.

Additional trials will also need to investigate duration of protection from anaphylaxis that BTKis confer after cessation of drug. Current data suggest that this period is relatively short, perhaps less than two days. In clinical trials, basophil activation and SPTs returned to baseline within one week after cessation of ibrutinib^20^. In humanized mice, acalabrutinib’s protection from anaphylaxis was no longer significant two days after the last dose^16^. These observations give insight into the turnover rate for BTK i*n vivo*. Studies in both humans and mice also demonstrate that BTKi onset of action is rapid, within hours to days. The rapid efficacy of BTKis in inhibiting mast cell and basophils raises the question of whether these drugs can also abort ongoing anaphylactic reactions, especially if administered together with epinephrine. Studies are currently underway in the authors’ laboratory to test the hypothesis that acalabrutinib can stop ongoing reactions and prevent mortality from anaphylaxis in a passive peanut allergy model in humanized mice.

### Food allergy:

Due to rapid onset of action and favorable safety profile with short-term courses, BTKis may be ideal therapies for intermittent use to prevent food-induced anaphylaxis in several clinical scenarios. One potential application for BTKis in food allergy would be as adjunct therapies for reducing or preventing adverse reactions during food oral immunotherapy (OIT). OIT can induce desensitization or a temporary state of hyporesponsiveness to foods in allergic patients^23^. The peanut-flour product Palforzia is currently the only FDA-approved OIT regimen, but numerous therapies for a variety of foods are in clinical trials. Upon reaching maintenance dose, OIT patients are generally protected from clinical reaction in the event of accidental food exposure. The doses of food OIT themselves, however, can also cause reactions that include anaphylaxis, especially during the build-up phase, such that a large portion of food-allergic patients ultimately fail to reach OIT maintenance due to recurrent reactions during build-up^25,26^. While anti-IgE therapies including omalizumab are under investigation as adjunct therapies for food OIT, omalizumab unfortunately requires several months to gain efficacy^25–27^. Concurrent BTKi administration may solve some of these ongoing challenges of food OIT. Short courses of BTKis may reduce the frequency and/or severity of adverse reactions to OIT doses during build-up, allowing patients to achieve maintenance dose. Adjunct use of BTKis may also facilitate shorter build-up times; for example, a two-day BTKi course could be administered at OIT onset to allow patients to rapidly achieve maintenance dose without adverse reactions, and could be discontinued later once allergen hyporesponsiveness is achieved to avoid interfering with long-term generation of protective allergen-specific IgG responses. While not yet demonstrated in humans *in vivo*, *in vitro* and animal studies have shown that inhibitors of essential kinases, including BTK and spleen-tyrosine kinase (SYK), can prevent IgE-mediated activation without interfering with the allergen desensitization process on a cellular level^28,29^.

One potential obstacle in using BTKis to facilitate food OIT is the target age group for this intervention. Children, especially young children, are more likely to achieve both desensitization during OIT and long-term remission after therapy is stopped^30^. However, safety data for the use of BTKis in children is limited, especially for newer BTKis. Currently, the only BTKi approved for use in children is ibrutinib, which is indicated for the treatment of cGVHD in children as young as one year of age and has demonstrated a safety profile in this age group consistent with other ibrutinib trials^31^. These safety results provide hope that newer BTKis in development would also be acceptable for use in children, even for chronic use. With favorable risk-benefit ratios, BTKis could potentially be used chronically for food allergy, especially in adult patients with life-threatening food allergies or those for whom food avoidance poses significant challenges. For children, however, chronic use of BTKis to prevent acute food reactions may not be desirable, as it remains unknown if inhibiting BTK chronically in food-allergic children would prohibit B cell production of protective allergen-specific IgG and thus impede the natural development of tolerance. This may be an acceptable risk in adults, who are unlikely to “outgrow” their food allergies even without BTKi treatment.

### Drug allergy:

Paralleling their proposed use in preventing IgE-mediated anaphylaxis to foods, BTKis show immense promise for mediating safe drug desensitizations. While no therapies specific for drug allergy have currently been approved, a drug desensitization involving closely monitored exposure to up-titrated doses may be considered for patients exhibiting hypersensitivity reactions to clinically essential medications. Desensitization procedures, however, are expensive, highly labor-intensive, and not accessible or performed at many hospitals and health centers that lack appropriate infrastructure such as an intensive care unit or an Allergy and Immunology consult service^32^. Because BTKis exhibit potential to consistently prevent IgE-mediated anaphylaxis with rapid onset, it is plausible that they may similarly reduce risk of breakthrough reactions during desensitizations. Thus, accessibility to drug desensitizations may expand into perhaps even diverse outpatient settings in the future.

A recent case report describes the successful use of ibrutinib in preventing severe breakthrough reactions during rapid drug desensitization to brentuximab vedotin (BV) and gemcitabine chemoimmunotherapy^33^. A 50-year-old male patient treated with BV/gemcitabine for refractory stage IVB Hodgkin lymphoma experienced severe anaphylaxis requiring epinephrine during his second treatment cycle, later characterized as BV-induced IgE-mediated hypersensitivity by positive intradermal and basophil activation tests. The patient failed initial BV desensitization with aspirin and montelukast pretreatment, developed hypotension, generalized pruritis, and flushing, and required vasopressors and cessation of the desensitization for clinical stabilization. Prior to the second desensitization, the patient was given two oral 420 mg doses of ibrutinib, and tolerated the desensitization with only generalized urticaria. With ibrutinib pretreatment, the desensitization duration was also decreased from 12 hours and 20 minutes to 6 hours and 54 minutes, suggestive of lower labor and cost burdens with BTKi use^33^. Randomized, placebo-controlled trials must be conducted to validate these preliminary data, but nonetheless, BTKis remain promising candidates to reduce the severity and/or frequency of breakthrough reactions during drug desensitizations.

Of note, because BTK is thought to be involved in only IgE-dependent hypersensitivity reactions, prophylactic BTKi use is unlikely to prevent IgE-independent reactions mediated by other receptor types or by non-specific mast cell activation. Examples include MRGPRX2 ligands, iodinated radiocontrast media, complement-mediated reactions, and cytokine-release reactions in response to some biologics^34–38^. The scope of BTKi use during drug desensitizations is therefore limited to prevention of immediate hypersensitivity reactions during desensitizations to medications with known IgE-mediated mechanisms, such as beta-lactam antibiotics and platinum-based chemotherapy agents^32,36^, or for individual cases wherein a patient demonstrates positive SPT, specific IgE, and/or basophil activation testing to the offending drug.

### Chronic urticaria:

BTK inhibition may be therapeutic for a number of allergic and/or autoimmune skin disorders, especially CSU, which encompasses features of both pathophysiological mechanisms^39^. One proposed mechanism for the pathogenesis of CSU centers around dysregulated FcεRI- and IgE-mediated signaling^2^. This is supported by presence of serum FcεRI-stimulating IgG autoantibodies and/or auto-allergic IgE in a subset of CSU patients, widely-reported success of CSU treatment with omalizumab, and positive correlations between omalizumab responsiveness and both serum IgE titers and basophil FcεRI expression^40^. Several recent clinical trials have investigated newer-generation, highly-selective BTKis as potential CSU therapies.

**Figure 3:**
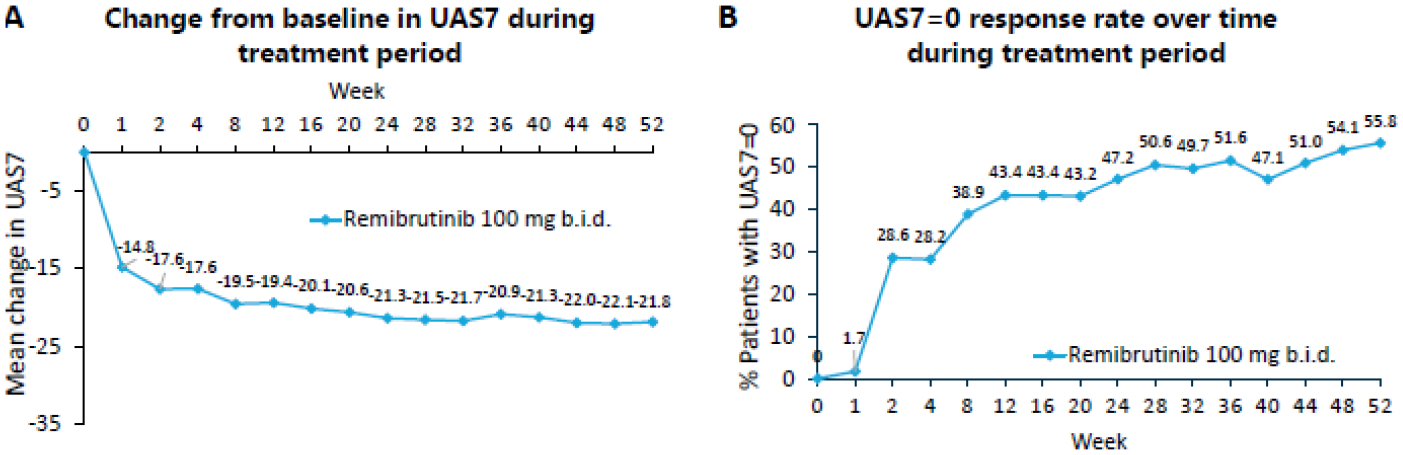
Remibrutinib induces a rapid and sustained improvement in UAS7 scores in CSU patients during long-term therapy up to 52 weeks. Remibrutinib 100 mg twice daily induced rapid and sustained improvement in UAS7 (A), and more than half of the patients achieved a UAS7 score of 0 at Week 52 of therapy (B). Figures adapted with permission from Jain et al^42^.

Remibrutinib is the furthest BTKi in development for CSU, demonstrating both remarkable efficacy and a promisingly favorable safety profile with chronic use^41,42^. In a phase 2a dose-selecting study, 309 adult subjects with CSU refractory to first-line second-generation H1 receptor antihistamines were randomly assigned to 12-week treatment arms with placebo, or remibrutinib 10 mg once daily, 35 mg once daily, 100 mg once daily, 10 mg twice daily, 25 mg twice daily, or 100 mg twice daily (NCT03926611). At week four, all remibrutinib arms demonstrated significant changes in Urticaria Activity Score Over 7 Days (UAS7) from baseline, with a change as much as −20.0 in patients taking 25 mg twice daily as compared to −5.4 for placebo^41^. Clinical improvement with remibrutinib was rapid, with onset as early as one week and robust regardless of prior responsiveness to omalizumab therapy^41,43^. Remibrutinib also showed a favorable safety profile, with a slight increased risk of bleeding events, all of which were minor cutaneous symptoms such as petechiae or purpura (6.7% in remibrutinib arms versus 2.4% in placebo). Important adverse events such as cytopenias and infections were similar between groups. These data suggest BTKis are effective in rapidly inhibiting FcεRI-mediated basophil and mast cell signaling in CSU patients. In the open-label extension of this phase 2b trial, 194 patients receiving 100 mg twice daily remibrutinib showed a −21.8 change in UAS7 at 52 weeks, with 55.8% and 68.0% of patients achieving complete remission (UAS7=0) and well-controlled disease (UAS7≤6), respectively^42^ (Figure 3). Safety data from the phase 2 extension showed similar findings as the core trial, apart from an increase in upper respiratory infections including SARS-CoV-2 due to remibrutinib in the extension compared to the core study placebo group, likely reflective of the fact that the extension study was carried out during peak pandemic years.

Most recently, the global, multi-center, randomized phase 3 REMIX-1 (NCT05030311) and REMIX-2 (NCT05032157) trials are ongoing in parallel, with an aggregated 613 patients receiving lower-dose 25 mg twice daily remibrutinib for up to 52 weeks and 312 patients receiving placebo for up to 24 weeks. Interim analysis presented at the American College of Allergy, Asthma, and Immunology 2023 annual meeting demonstrated that as compared to placebo, remibrutinib-treated patients experienced significant decreases in UAS7 at 12 weeks (−20.1) from baseline, albeit with slightly lower percentages of remibrutinib-receiving patients achieving complete remission (29.5% across both REMIX-1 and −2) or well-controlled disease (48.8%) at week 12 compared to the same dose in the phase 2 trial^44^.

**Figure 4:**
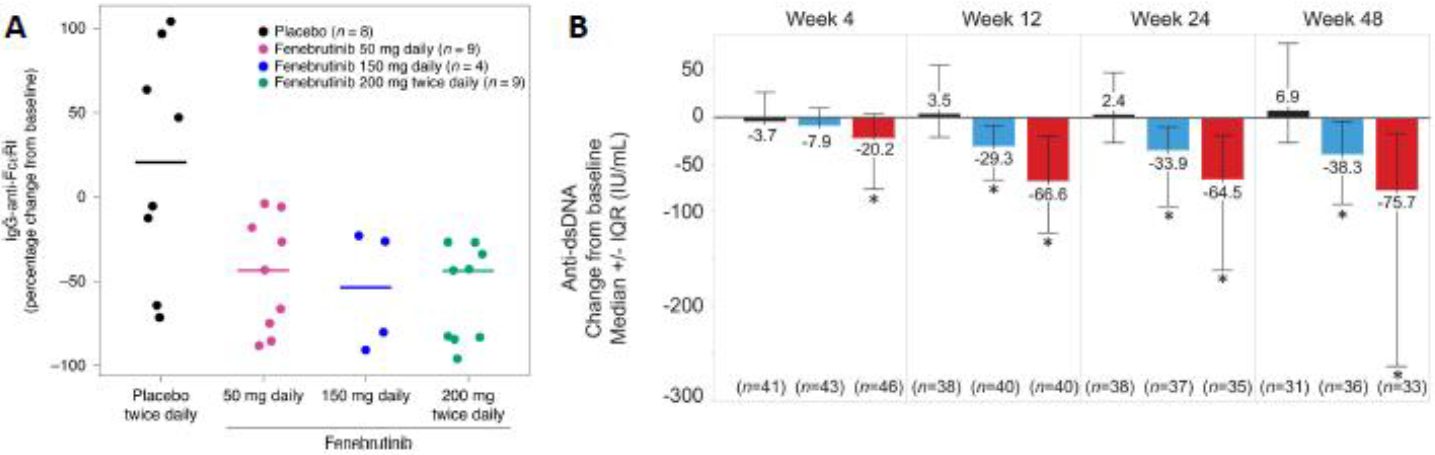
Fenebrutinib reduces pathogenic specific IgG titers over time. (A) In addition to strong clinical efficacy in CSU, all fenebrutinib doses (red, blue, and green dots) reduced serum levels of IgG-anti-FcεRI autoantibodies compared to placebo (black dots) at 8 weeks after initiation of treatment in its phase 2 trial. Figure reproduced with permission from Metz et al^45^. (B) In its phase 2 trial in SLE patients, fenebrutinib 150 mg daily (blue bars) and 200 mg twice daily (red bars) both demonstrated reduction in serum IgG-anti-dsDNA autoantibodies compared to placebo (black bars)^46^. Bars show the median and interquartile range. * P < 0.05 compared to placebo.

In addition to rapid efficacy due to suppression of FcεRI-dependent activation of mast cells, chronic BTKi therapy for CSU may have long-term efficacy that is attributed in part to long-term reduction of autoantibody titers due to suppression of autoreactive B cell development. Though autoantibody titer data have not been released from remibrutinib trials, data from trials with the highly-selective, noncovalent BTKi fenebrutinib (Genentech) indicate a clear trend. In a phase 2a trial for CSU (NCT03137069), adult subjects treated with fenebrutinib achieved the primary endpoint of decreased UAS7 score coupled with substantially decreased autoantibody titers at eight weeks compared to placebo^45^ (Figure 4A). Paralleling these findings, fenebrutinib treatment for at least 12 weeks also significantly decreased IgG autoantibody and rheumatoid factor titers in rheumatoid arthritis patients and anti-dsDNA autoantibodies in systemic erythematous lupus patients^46,47^ (Figure 4B). Though fenebrutinib is no longer in development for CSU due to transient elevations in liver transaminases during its trials for both CSU and autoimmune diseases^45–47^, similar reductions in autoantibody titers could be expected with other BTKis.

Other BTKis are also entering the chronic urticaria space. Rilzabrutinib’s phase 2 trial in H1-antihistamine-refractory CSU in adults has two parts: a double-blinded, four-arm, 12-week treatment period followed by 40-week open-label extension (NCT05107115). Interim results from part 1 were presented at the American Academy of Allergy, Asthma, and Immunology 2024 Annual Meeting. Rilzabrutinib induced a rapid and significant response, with a change in UAS7 from baseline (−14.8) in patients treated with 400 mg three times daily compared to placebo (−8.07)^48^. The most common adverse events in this dosing arm were diarrhea (29.3%), nausea (19.5%), and headache (9.8%) compared to placebo (15.0, 5.0, and 0%, respectively). Importantly, no instances of bleeding, cytopenias, or atrial fibrillation were observed in any treatment arm. Rilzabrutinib is also being investigated in numerous phase 2 and 3 trials for various rheumatologic and autoimmune diseases and has shown efficacy in immune-mediated thrombocytopenia^49,50^.

### Atopic dermatitis:

Several phase 2 trials using next-generation BTKis have been recently completed for AD. A phase 2 randomized, double-blind, 2-arm, placebo-controlled trial was recently completed to evaluate rilzabrutinib’s safety and efficacy in adult patients with moderate-to-severe atopic dermatitis (NCT05018806). Atuzabrutinib (PRN473/SAR444727; Principia Pharma/Sanofi), another compound from Sanofi, is a novel topical formulation of BTKi studied in a phase 2a trial for mild-to-moderate AD (NCT04992546)^51^. Unfortunately, the atuzabrutinib program has since been discontinued and results are unavailable. Finally, branebrutinib (BMS-986166; Bristol-Myers Squibb) was investigated in a small randomized, double-blind, placebo-controlled phase 2 trial (*n*=17) for moderate-to-severe AD (NCT05014438), with results anticipated in 2024. As of the writing of this review, no phase 3 trials of BTKis in AD have been initiated.

### Asthma:

BTKis may be a potential adjuvant therapy for allergic asthma. Several preclinical studies propose that BTKis could synergize with beta-agonist and inhaled corticosteroid therapies to treat several asthma endotypes. A 2016 study found that a six-day course of novel aerosolized reversible BTKi RN983 decreases pulmonary inflammation, bronchial eosinophils, and airway resistance in a mouse model of ovalbumin-induced eosinophilic asthma, as compared to three-day inhaled budesonide treatment or single-dose salbutamol^18^. Additionally, because BTK is expressed in other leukocytes including neutrophils and types 2 and 3 innate lymphoid cells, BTKis may be effective for neutrophilic asthma. In a mouse model of cockroach allergen extract-induced mixed granulocytic asthma, a seven-day intranasal ibrutinib course not only decreased Th2/Th17 cytokine release and eosinophilic and lymphocytic airway inflammation to a greater extent at higher doses than did corticosteroids, but also attenuated typically corticosteroid-unresponsive neutrophilic airway inflammation at all tested ibrutinib doses^52^. Concurrent ibrutinib plus dexamethasone treatment was synergistic in further suppressing airway hyperreactivity, Th2 cytokine release, and eosinophil accumulation in these mice^53^.

Despite these promising preclinical results, however, the clinical benefit of BTKis for asthma treatment has thus been disappointing. A recent halted phase 2 randomized control trial observed no differences in forced expiratory volume (FEV1), Asthma Control Questionnaire (ACQ5) score, or short-acting beta agonist (SABA) usage in patients with inadequately-controlled asthma treated with 100 mg once daily oral remibrutinib as compared to placebo (NCT03944707). A phase 2 randomized control trial for adjunct treatment of moderate-to-severe asthma with rilzabrutinib is still ongoing outside of the United States (NCT05104892)^49^. Still, the landscape of BTKi use as adjunct asthma therapy remains dynamic as more mechanistic insights into mast cell physiology and asthma pathophysiology are uncovered.

### Other allergic disorders:

Additional allergic indications may also benefit from treatment with BTKis, though the exact potential in these spaces remains uncertain. Because BTK is not thought to play a role in signaling through IgE-independent receptors on mast cells or basophils, diseases that primarily involve these pathways or other non-specific methods of activating these cells would not likely respond to BTK inhibition. Likewise, targeting the FcεRI pathway may not be efficacious in disorders which display mast cell activation by unknown or non-specific triggers, including idiopathic mast cell activation syndrome, idiopathic anaphylaxis, and mastocytosis. For some IgE-mediated disorders, such as allergic rhinitis and conjunctivitis, systemic BTKis would likely have an unfavorable risk/benefit ratio. One could speculate, however, that topical versions in nasal spray or eyedrop form could effectively treat these conditions.

## SAFETY CONSIDERATIONS

Though cumulative safety data for next-generation BTKis have been positive^1,54^, clinicians’ safety concerns for pharmacologic BTK inhibitors may still be overshadowed by knowledge of the severe phenotype of congenital BTK deficiency, otherwise known as X-linked agammaglobulinemia (XLA). XLA is a primary immunodeficiency characterized by low or absent B cells and resulting lack of humoral immunity^55,56^. Fortunately, pharmacologic inhibition of BTK in adult patients does not appear to have much functional similarity to XLA. The most common side effects of currently-approved (first- and second-generation) BTKis tend to be mild in nature and include diarrhea, nausea, upper respiratory tract infections, and rash^9,57–60^. More serious but rare toxicities can occur in cancer patients taking currently-approved BTKis, including cytopenias, hypertension, bleeding, and cardiac arrhythmias^9,61–63^. Several of these adverse effects are thought to be due to off-target effects on TEC kinase and EGFR, especially for ibrutinib^61–64^.

In the context of BTK’s functions *in vivo*, infectious complications due to potential adverse effects on immunoglobulin production or even innate immune function are among the most feared side effects of these medications. There are mixed reports of early-generation BTKis causing increased risk of infections in patients with cancers including CLL, especially as CLL in itself increases patients’ risk of severe infectious complications. Perhaps counterintuitively, ibrutinib has been shown to restore some immune function in cancer patients, including preferential suppression of malignant B cells, restoration of T cell responses, and return of blood cell counts to normal ranges^65–67^. CLL patients experience a transient increase in IgM but sustained increase in IgA levels, while IgG levels appear relatively stable during ibrutinib therapy^68^. This general pattern holds true with more selective BTKis, including remibrutinib, fenebrutinib, and evobrutinib, which do not appear to cause hypogammaglobulinemia^42,46,54^. In its CSU phase 2 extension, remibrutinib modestly reduced total IgG levels by a mean of 0.534 g/L from a baseline of 11.043 g/L in patients taking it for 52 weeks^29^. Similarly, IgM was slightly decreased by 0.13 g/L from a baseline of 1.047 g/L, and IgE levels decreased by 140.22 mg/L from a baseline of 839.47 mg/L. Remibrutinib did not affect IgA levels. The corresponding effects of BTKis on vaccine responses is unclear given the lack of rigorous studies and the presence of confounding variables, such as a B cell malignancy or administration of multiple chemotherapies at once. One study in 81 patients demonstrated that CLL patients on ibrutinib or acalabrutinib monotherapy do mount SARS-CoV-2 vaccine responses: seroconversion was detected in 53% of BTKi-treated patients (*n*=54), 43% of patients on single-agent venetoclax, and 75% of treatment-naïve CLL patients^69^. Of patients on a BTKi, those who interrupted therapy for a mean of approximately two weeks had similar post-booster seroconversion rates (83%) compared to those who did not interrupt BTKi therapy (82%), though patients who had paused BTKi therapy exhibited higher anti-spike protein antibody titers^69^. The next-generation BTKi evobrutinib (Merck), however, did not affect immune responses to SARS-CoV-2 vaccines compared to placebo in a post-hoc analysis of its phase 2 trial for multiple sclerosis^70^.

Infection rates from BTKis also appears to vary considerably based on the particular medication, with ibrutinib having the most severe infectious profile and newer-generation medications having less severe complications^9,71^. Infectious events during ibrutinib therapy can include bacterial as well as viral infections, including reactivation of HSV and VZV^72,73^. Of most concern, ibrutinib therapy increases the risk of invasive fungal infections, typically occurring about four to six months after starting therapy, and primarily in CLL patients rather than Waldenström’s macroglobulinemia (WM) or other malignancies^71,73–75^. The mechanisms are unclear, but unlike newer BTKis, it is hypothesized that ibrutinib also inhibits T cell function due to off-target activity on ITK.

Fortunately, the next-generation BTKis are not associated with fungal infections, but they do have several on-target immune effects that should be taken into consideration. Aside from B cells, BTK has important roles in several other immune pathways and functions, including NLRP3 inflammasome activation, inflammatory cytokine production in macrophages, and signaling through multiple Toll-like receptors^76^. Cytopenias, especially neutropenia, have been reported with early BTKis, but are not as prevalent in next-generation BTKis in clinical trials. In the remibrutinib extension, 1.0% of patients on remibrutinib had a cytopenia, compared to 2.4% of patients on placebo in the core study^42^. Cytopenias in remibrutinib-treated patients were mild and not associated with infections. Additionally, there are reports of hepatitis B reactivation in patients taking ibrutinib and acalabrutinib, suggesting that checking for latent infection is warranted for those particular drugs^77^. Despite their ability to cause rare infectious complications, BTKis are also currently under investigation as adjunct agents for protection against cytokine release storm during SARS-CoV-2 infection^78^. Several studies have shown that BTK inhibition may protect against pulmonary injury and even improve pulmonary function in hypoxic patients with SARS-CoV-2 infection^79–82^. Together, these data suggest that many of the more severe infectious complications from BTKi therapy may be a result of either off-target effects and/or a sequela of malignancy rather than from the treatment itself. Perhaps BTKis can exacerbate preexisting dysfunctional immune responses to foreign antigens in immunocompromised patients with leukemia or lymphoma, while having far less impact on immune function in patients with normal B cells.

Resistance to BTKis targeting the C-481 domain has been reported in patients taking BTKis chronically for cancers^83^. Mechanisms of resistance largely involve malignant B cell mutations in the BTK kinase domain as well as downstream PLCγ2 mutations that can bypass BTK inhibition^83^. Though it is theoretically possible that normal and non-malignant mast cells and basophils could develop resistance, this seems unlikely given the lack of selective pressure, as BTK-dependent pathways are not essential for mast cell or basophil survival. There are no data to suggest that BTKis deplete either wildtype cell type *in vivo*. Though prior studies have demonstrated that ibrutinib reduces the survival of canine neoplastic mast cells^84^, our laboratory’s own unpublished data have not found any effects of chronic exposure to several different BTKis, including ibrutinib, tirabrutinib, or acalabrutinib, on the survival of primary human skin-derived mast cells in culture.

## CONCLUSION

BTK inhibitors show incredible promise in treating several allergic disorders, especially as newer BTKis demonstrate higher selectivity for BTK and fewer side effects than first-generation compounds. Their ability to abrogate signaling through the FcεRI pathway allows them to act in an allergen-independent manner. Furthermore, rapid onset of action and transient efficacy *in vivo* make BTKis ideal short-term therapies for food or drug desensitizations. Indeed, recent trial results demonstrate the exciting application of BTKis to prevent IgE-mediated anaphylaxis to foods, although additional clinical trials are needed to determine optimal dosing, duration of protection, and factors predictive of efficacy. In addition, other trials have demonstrated efficacy and safety with chronic usage of next-generation BTKis for chronic urticaria. Finally, BTKis may also be effective in disorders such as asthma in which leukocytes other than mast cells and basophils are involved, though clinical trials are needed to demonstrate BTKi utility and safety for these indications. Thus, the landscape of BTKi therapies remains promising, particularly as newer BTKi iterations continue to minimize toxicity while maintaining efficacy.

## Figures and Tables

**Table 1:** BTK inhibitors that are FDA-approved and in clinical trials for allergic disorders

	Medication	FDA-Approvals	IC_50_ (nM)	Selectivity for BTK	Pharmacology	Trials in Allergy/Immunology
1^st^ generation	Ibrutinib	2013: MCL 2014: CLL 2015: WM 2016: SLL 2017: MZL and GVHD	0.5	Moderate – off-target effects on BMX, EGFR, ErbB2, ErbB4, ITK, JAK3, TEC, and TXK kinases	Type of inhibitor: Covalent Half-life: 4–6 hours Dose: 420 mg orally once daily	Food allergy: Phase 2: NCT03149315^20^
2^nd^ generation	Acalabrutinib	2017: MCL 2019: CLL/SLL	5.1	Moderate – lower off-target activity on BMX, ErbB4, TEC, and TXK than ibrutinib	Type of inhibitor: Covalent Half-life: 1.4 hours (6.4 hours for its active metabolite) Dose: 100 mg orally twice daily	Food allergy: Phase 2: NCT05038904^19^
	Zanubrutinib	2019: CLL and MCL 2021: MZL and WM	0.5	Moderate – lower off-target activity on ITK, EGFR, and JAK3 than ibrutinib	Type of inhibitor: Covalent Half-life: 2–4 hours Dose: 160 mg orally twice daily or 320 mg once daily	None
	Pirtobrutinib	2023: BTKi refractory MCL, CLL, and SLL	0.85	Moderate to high	Type of inhibitor: Non-covalent (binds to both WT and mutant BTK) Half-life: 19 hours Dose: 200 mg orally once daily	None
Next generation	Remibrutinib (LOU064, Novartis)	Not yet approved	1.3	High	Type of inhibitor: Covalent Half-life: 1–2 hours Dose: 100 mg orally twice daily	CSU: Phase 2b: NCT03926611^41^ Phase 2b extension: NCT04109313^42^ Phase 3: NCT05030311 (ongoing)^44^ Phase 3 extension: NCT05513001 (ongoing) Food allergy: Phase 2: NCT05432388 (ongoing) Asthma: Phase 2: NCT03944707 (terminated)
	Rilzabrutinib (PRN1008/)	Not yet approved	1.3	High	Type of inhibitor: Covalent Half-life: 2.45 hours Dose: Undetermined	CSU: Phase 2: NCT05107115 (ongoing) AD: Phase 2: NCT05018806 (completed) Asthma: Phase 2: NCT05104892 (ongoing)
	Atuzabrutinib (PRN473 / SAR444727; Principia Pharma/Sanofi)	Not approved (development program halted)	1.8	High	Type of inhibitor: Covalent Half-life: n/a (topical inhibitor) Dose: Undetermined	AD: Phase 2: NCT04992546 (completed)
	Branebrutinib (BMS-986166; Bristol-Myers Squibb)	Not yet approved	0.1	High	Type of inhibitor: Covalent Half-life: 7–11 hours Dose: Undetermined	AD: Phase 2: NCT05014438 (completed)
	Fenebrutinib	Not yet approved	WT: 0.9 Mutant: 1.6	High	Type of inhibitor: Non-covalent Half-life: 6.1–11 hours Dose: Undetermined	CSU (No longer in development for CSU) Phase 2a: NCT03137069^45^ Phase 2b: NCT03693625 (terminated)

**Abbreviations:** AD, atopic dermatitis; CLL, chronic lymphocytic leukemia; CSU, chronic spontaneous urticaria; GVHD, graft-versus-host disease; IC50, half-maximal inhibitory concentration; MCL, mantle cell lymphoma; MZL, marginal zone lymphoma; WM, Waldenström’s macroglobulinemia; WT, wild-type

## Key Messages:

- Pharmacologic inhibitors of Bruton’s tyrosine kinase (BTK) can fully prevent IgE-mediated activation of human mast cells and basophils in an allergen-independent manner.
- BTK inhibitors have a rapid onset of action *in vivo* and transient efficacy, making them potentially useful short-term adjunct therapies to facilitate food or drug desensitizations.
- A two-day course of the second-generation BTK inhibitor acalabrutinib has been shown to reduce or completely prevent clinical reactivity to allergenic foods in adults during oral food challenge, with an encouraging safety profile for short-term use.
- Remibrutinib is a next-generation BTK inhibitor in development for CSU which has excellent clinical efficacy and a favorable safety profile for chronic use.
- Future clinical trials will further elucidate the minimum effective dose and utility of using BTK inhibitors for the treatment of allergic disorders.

## ABBREVIATIONS USED:

AD: atopic dermatitis
BCR: B cell receptor
BTK: Bruton’s tyrosine kinase
BTKi: BTK inhibitor
cGVHD: chronic graft-versus-host-disease
CLL: chronic lymphocytic leukemia
CSU: chronic spontaneous urticaria
GVHD: graft-versus-host disease
IC50: half-maximal inhibitory concentration
IgE: immunoglobulin E
MCL: mantle cell lymphoma
MZL: marginal zone lymphoma
OIT: oral immunotherapy
UAS7: Urticaria Activity Score Over 7 Days
WM: Waldenströms macroglobulinemia
XLA: X-linked agammaglobulinemia
